# Supplementary material for: Modeling glioblastoma heterogeneity as a dynamic network of cell states
Source: Mol Syst Biol. 2021 Sep 16;17(9):e10105. doi: 10.15252/msb.202010105 (PMC8444284; doi:10.15252/msb.202010105)
Supplement: Supplementary file 5 — Source Data for Figure 3 [file MSB-17-e10105-s001.zip › Figure3A_sourcedata/GSEA_3065/hallmarks_state5.GseaPreranked.1623416302988/HALLMARK_E2F_TARGETS.html]

Details for gene set HALLMARK\_E2F\_TARGETS[GSEA]

|  || Dataset | state5 |
| Phenotype | NoPhenotypeAvailable |
| Upregulated in class | na\_neg |
| GeneSet | HALLMARK\_E2F\_TARGETS |
| Enrichment Score (ES) | -0.4096345 |
| Normalized Enrichment Score (NES) | -1.5168757 |
| Nominal p-value | 0.0011806375 |
| FDR q-value | 0.041821253 |
| FWER p-Value | 0.342 |
Table: GSEA Results Summary

  

Fig 1: Enrichment plot: HALLMARK\_E2F\_TARGETS      
 Profile of the Running ES Score & Positions of GeneSet Members on the Rank Ordered List

  

| PROBE | GENE SYMBOL | GENE\_TITLE | RANK IN GENE LIST | RANK METRIC SCORE | RUNNING ES | CORE ENRICHMENT || 1 | LUC7L3 |  |  | 65 | 0.589 | 0.0108 | No |
| 2 | PRDX4 |  |  | 80 | 0.553 | 0.0263 | No |
| 3 | PNN |  |  | 195 | 0.442 | 0.0270 | No |
| 4 | LBR |  |  | 198 | 0.440 | 0.0404 | No |
| 5 | HELLS |  |  | 280 | 0.393 | 0.0433 | No |
| 6 | GINS4 |  |  | 492 | 0.314 | 0.0290 | No |
| 7 | NUP107 |  |  | 507 | 0.309 | 0.0370 | No |
| 8 | BRCA1 |  |  | 522 | 0.305 | 0.0448 | No |
| 9 | TFRC |  |  | 727 | 0.255 | 0.0295 | No |
| 10 | ORC6 |  |  | 806 | 0.240 | 0.0281 | No |
| 11 | WEE1 |  |  | 919 | 0.222 | 0.0222 | No |
| 12 | XPO1 |  |  | 933 | 0.220 | 0.0275 | No |
| 13 | WDR90 |  |  | 1034 | 0.206 | 0.0225 | No |
| 14 | PRKDC |  |  | 1051 | 0.204 | 0.0270 | No |
| 15 | MMS22L |  |  | 1126 | 0.195 | 0.0246 | No |
| 16 | NASP |  |  | 1225 | 0.182 | 0.0191 | No |
| 17 | ILF3 |  |  | 1314 | 0.170 | 0.0144 | No |
| 18 | KIF18B |  |  | 1411 | 0.161 | 0.0084 | No |
| 19 | PMS2 |  |  | 1585 | 0.142 | -0.0068 | No |
| 20 | BRCA2 |  |  | 1781 | 0.128 | -0.0251 | No |
| 21 | PRIM2 |  |  | 1881 | 0.120 | -0.0326 | No |
| 22 | PDS5B |  |  | 1989 | 0.112 | -0.0413 | No |
| 23 | SMC6 |  |  | 1991 | 0.112 | -0.0379 | No |
| 24 | CIT |  |  | 2076 | 0.107 | -0.0442 | No |
| 25 | EED |  |  | 2123 | 0.104 | -0.0462 | No |
| 26 | MXD3 |  |  | 2178 | 0.100 | -0.0493 | No |
| 27 | TRA2B |  |  | 2285 | 0.093 | -0.0584 | No |
| 28 | NUP205 |  |  | 2306 | 0.091 | -0.0579 | No |
| 29 | SRSF1 |  |  | 2334 | 0.090 | -0.0582 | No |
| 30 | DIAPH3 |  |  | 2387 | 0.086 | -0.0614 | No |
| 31 | NOP56 |  |  | 2390 | 0.086 | -0.0590 | No |
| 32 | MELK |  |  | 2402 | 0.085 | -0.0576 | No |
| 33 | POLE |  |  | 2438 | 0.083 | -0.0590 | No |
| 34 | ATAD2 |  |  | 2545 | 0.077 | -0.0687 | No |
| 35 | RNASEH2A |  |  | 2560 | 0.076 | -0.0679 | No |
| 36 | MRE11 |  |  | 2756 | 0.065 | -0.0881 | No |
| 37 | AURKB |  |  | 2853 | 0.060 | -0.0972 | No |
| 38 | CHEK1 |  |  | 2877 | 0.059 | -0.0980 | No |
| 39 | RAD51AP1 |  |  | 2905 | 0.057 | -0.0992 | No |
| 40 | SRSF2 |  |  | 2908 | 0.057 | -0.0977 | No |
| 41 | STAG1 |  |  | 2972 | 0.054 | -0.1032 | No |
| 42 | LIG1 |  |  | 3181 | 0.042 | -0.1255 | No |
| 43 | CCP110 |  |  | 3202 | 0.041 | -0.1265 | No |
| 44 | CTPS1 |  |  | 3314 | 0.036 | -0.1381 | No |
| 45 | BUB1B |  |  | 3355 | 0.034 | -0.1416 | No |
| 46 | ING3 |  |  | 3421 | 0.031 | -0.1480 | No |
| 47 | MLH1 |  |  | 3605 | 0.023 | -0.1681 | No |
| 48 | SHMT1 |  |  | 3695 | 0.020 | -0.1776 | No |
| 49 | SMC1A |  |  | 3696 | 0.020 | -0.1769 | No |
| 50 | SPC24 |  |  | 3726 | 0.019 | -0.1796 | No |
| 51 | RFC3 |  |  | 3863 | 0.013 | -0.1947 | No |
| 52 | ORC2 |  |  | 3888 | 0.012 | -0.1970 | No |
| 53 | DLGAP5 |  |  | 3907 | 0.011 | -0.1987 | No |
| 54 | NCAPD2 |  |  | 3914 | 0.011 | -0.1991 | No |
| 55 | CCNE1 |  |  | 4201 | -0.001 | -0.2316 | No |
| 56 | GINS1 |  |  | 4224 | -0.002 | -0.2340 | No |
| 57 | POLD1 |  |  | 4333 | -0.007 | -0.2460 | No |
| 58 | TACC3 |  |  | 4450 | -0.012 | -0.2589 | No |
| 59 | DONSON |  |  | 4454 | -0.012 | -0.2588 | No |
| 60 | RBBP7 |  |  | 4521 | -0.015 | -0.2659 | No |
| 61 | MCM4 |  |  | 4534 | -0.015 | -0.2667 | No |
| 62 | KIF2C |  |  | 4621 | -0.019 | -0.2759 | No |
| 63 | TCF19 |  |  | 4635 | -0.020 | -0.2768 | No |
| 64 | MYC |  |  | 4656 | -0.020 | -0.2784 | No |
| 65 | CDK1 |  |  | 4766 | -0.025 | -0.2901 | No |
| 66 | BARD1 |  |  | 4776 | -0.025 | -0.2903 | No |
| 67 | CENPE |  |  | 4787 | -0.025 | -0.2907 | No |
| 68 | MCM2 |  |  | 4824 | -0.027 | -0.2940 | No |
| 69 | HMMR |  |  | 4826 | -0.027 | -0.2932 | No |
| 70 | EZH2 |  |  | 4858 | -0.028 | -0.2959 | No |
| 71 | NUP153 |  |  | 4929 | -0.030 | -0.3029 | No |
| 72 | DSCC1 |  |  | 4941 | -0.031 | -0.3032 | No |
| 73 | DDX39A |  |  | 4948 | -0.031 | -0.3029 | No |
| 74 | MCM7 |  |  | 4986 | -0.032 | -0.3061 | No |
| 75 | CNOT9 |  |  | 5053 | -0.035 | -0.3126 | No |
| 76 | PSMC3IP |  |  | 5222 | -0.041 | -0.3304 | No |
| 77 | SPAG5 |  |  | 5225 | -0.041 | -0.3293 | No |
| 78 | POLA2 |  |  | 5229 | -0.042 | -0.3284 | No |
| 79 | TP53 |  |  | 5275 | -0.043 | -0.3322 | No |
| 80 | TBRG4 |  |  | 5300 | -0.044 | -0.3335 | No |
| 81 | SPC25 |  |  | 5312 | -0.045 | -0.3334 | No |
| 82 | NUDT21 |  |  | 5349 | -0.047 | -0.3360 | No |
| 83 | CCNB2 |  |  | 5382 | -0.048 | -0.3382 | No |
| 84 | POLD3 |  |  | 5393 | -0.048 | -0.3378 | No |
| 85 | PPP1R8 |  |  | 5401 | -0.048 | -0.3371 | No |
| 86 | CDC25A |  |  | 5443 | -0.050 | -0.3403 | No |
| 87 | SMC3 |  |  | 5536 | -0.054 | -0.3490 | No |
| 88 | CTCF |  |  | 5558 | -0.055 | -0.3497 | No |
| 89 | TRIP13 |  |  | 5570 | -0.056 | -0.3492 | No |
| 90 | MSH2 |  |  | 5621 | -0.059 | -0.3531 | No |
| 91 | TIMELESS |  |  | 5717 | -0.063 | -0.3620 | No |
| 92 | TOP2A |  |  | 5722 | -0.063 | -0.3605 | No |
| 93 | CDCA3 |  |  | 5726 | -0.063 | -0.3589 | No |
| 94 | KIF22 |  |  | 5758 | -0.064 | -0.3604 | No |
| 95 | IPO7 |  |  | 5792 | -0.065 | -0.3622 | No |
| 96 | KPNA2 |  |  | 5811 | -0.066 | -0.3622 | No |
| 97 | CDCA8 |  |  | 5863 | -0.068 | -0.3659 | No |
| 98 | RACGAP1 |  |  | 5902 | -0.070 | -0.3680 | No |
| 99 | NBN |  |  | 5977 | -0.073 | -0.3742 | No |
| 100 | CDKN1B |  |  | 5996 | -0.074 | -0.3739 | No |
| 101 | RFC1 |  |  | 6011 | -0.074 | -0.3732 | No |
| 102 | EXOSC8 |  |  | 6020 | -0.075 | -0.3718 | No |
| 103 | PPM1D |  |  | 6025 | -0.076 | -0.3699 | No |
| 104 | CSE1L |  |  | 6160 | -0.083 | -0.3826 | No |
| 105 | PAICS |  |  | 6198 | -0.085 | -0.3842 | No |
| 106 | MKI67 |  |  | 6211 | -0.086 | -0.3829 | No |
| 107 | RRM2 |  |  | 6308 | -0.091 | -0.3910 | No |
| 108 | HUS1 |  |  | 6314 | -0.091 | -0.3888 | No |
| 109 | ZW10 |  |  | 6365 | -0.093 | -0.3916 | No |
| 110 | CENPM |  |  | 6368 | -0.093 | -0.3889 | No |
| 111 | ASF1B |  |  | 6400 | -0.095 | -0.3895 | No |
| 112 | DNMT1 |  |  | 6455 | -0.099 | -0.3926 | No |
| 113 | UNG |  |  | 6479 | -0.100 | -0.3921 | No |
| 114 | LYAR |  |  | 6513 | -0.102 | -0.3927 | No |
| 115 | SMC4 |  |  | 6517 | -0.102 | -0.3899 | No |
| 116 | KIF4A |  |  | 6529 | -0.103 | -0.3879 | No |
| 117 | MCM5 |  |  | 6553 | -0.105 | -0.3873 | No |
| 118 | PLK1 |  |  | 6581 | -0.107 | -0.3871 | No |
| 119 | MAD2L1 |  |  | 6600 | -0.108 | -0.3857 | No |
| 120 | DCK |  |  | 6637 | -0.110 | -0.3864 | No |
| 121 | LMNB1 |  |  | 6638 | -0.110 | -0.3830 | No |
| 122 | ANP32E |  |  | 6655 | -0.111 | -0.3814 | No |
| 123 | RPA2 |  |  | 6705 | -0.114 | -0.3835 | No |
| 124 | DEPDC1 |  |  | 6723 | -0.115 | -0.3818 | No |
| 125 | UBR7 |  |  | 6772 | -0.119 | -0.3836 | No |
| 126 | PRPS1 |  |  | 6774 | -0.119 | -0.3800 | No |
| 127 | RAD1 |  |  | 6786 | -0.120 | -0.3776 | No |
| 128 | CDC20 |  |  | 6985 | -0.136 | -0.3959 | No |
| 129 | RAD51C |  |  | 6989 | -0.136 | -0.3920 | No |
| 130 | AURKA |  |  | 6990 | -0.136 | -0.3878 | No |
| 131 | TIPIN |  |  | 6997 | -0.137 | -0.3843 | No |
| 132 | CDC25B |  |  | 7102 | -0.146 | -0.3916 | No |
| 133 | ASF1A |  |  | 7155 | -0.151 | -0.3928 | No |
| 134 | TMPO |  |  | 7296 | -0.167 | -0.4036 | No |
| 135 | SYNCRIP |  |  | 7350 | -0.175 | -0.4042 | Yes |
| 136 | EIF2S1 |  |  | 7366 | -0.177 | -0.4005 | Yes |
| 137 | MCM6 |  |  | 7375 | -0.178 | -0.3959 | Yes |
| 138 | TK1 |  |  | 7377 | -0.178 | -0.3905 | Yes |
| 139 | USP1 |  |  | 7398 | -0.181 | -0.3872 | Yes |
| 140 | RPA1 |  |  | 7418 | -0.183 | -0.3837 | Yes |
| 141 | PSIP1 |  |  | 7460 | -0.187 | -0.3825 | Yes |
| 142 | MTHFD2 |  |  | 7486 | -0.190 | -0.3795 | Yes |
| 143 | MCM3 |  |  | 7552 | -0.198 | -0.3808 | Yes |
| 144 | UBE2T |  |  | 7561 | -0.199 | -0.3756 | Yes |
| 145 | BIRC5 |  |  | 7596 | -0.204 | -0.3731 | Yes |
| 146 | AK2 |  |  | 7671 | -0.213 | -0.3750 | Yes |
| 147 | NOLC1 |  |  | 7699 | -0.218 | -0.3713 | Yes |
| 148 | RAD21 |  |  | 7785 | -0.231 | -0.3738 | Yes |
| 149 | PHF5A |  |  | 7818 | -0.238 | -0.3701 | Yes |
| 150 | TUBG1 |  |  | 7846 | -0.243 | -0.3657 | Yes |
| 151 | CKS2 |  |  | 7972 | -0.270 | -0.3715 | Yes |
| 152 | HMGB2 |  |  | 7973 | -0.271 | -0.3631 | Yes |
| 153 | MYBL2 |  |  | 8041 | -0.283 | -0.3620 | Yes |
| 154 | RFC2 |  |  | 8049 | -0.285 | -0.3540 | Yes |
| 155 | CDKN3 |  |  | 8079 | -0.293 | -0.3482 | Yes |
| 156 | PCNA |  |  | 8082 | -0.293 | -0.3394 | Yes |
| 157 | CDKN2C |  |  | 8091 | -0.295 | -0.3312 | Yes |
| 158 | DUT |  |  | 8118 | -0.300 | -0.3249 | Yes |
| 159 | CBX5 |  |  | 8171 | -0.318 | -0.3209 | Yes |
| 160 | SSRP1 |  |  | 8178 | -0.320 | -0.3117 | Yes |
| 161 | CKS1B |  |  | 8241 | -0.336 | -0.3084 | Yes |
| 162 | GSPT1 |  |  | 8299 | -0.355 | -0.3039 | Yes |
| 163 | DCTPP1 |  |  | 8303 | -0.356 | -0.2932 | Yes |
| 164 | PA2G4 |  |  | 8320 | -0.360 | -0.2839 | Yes |
| 165 | XRCC6 |  |  | 8325 | -0.361 | -0.2732 | Yes |
| 166 | SLBP |  |  | 8396 | -0.387 | -0.2692 | Yes |
| 167 | NAP1L1 |  |  | 8404 | -0.389 | -0.2580 | Yes |
| 168 | HMGB3 |  |  | 8427 | -0.399 | -0.2482 | Yes |
| 169 | HNRNPD |  |  | 8436 | -0.402 | -0.2366 | Yes |
| 170 | PTTG1 |  |  | 8446 | -0.406 | -0.2251 | Yes |
| 171 | NAA38 |  |  | 8458 | -0.413 | -0.2136 | Yes |
| 172 | POLD2 |  |  | 8508 | -0.439 | -0.2056 | Yes |
| 173 | POLE4 |  |  | 8536 | -0.451 | -0.1947 | Yes |
| 174 | RAN |  |  | 8555 | -0.463 | -0.1825 | Yes |
| 175 | RPA3 |  |  | 8572 | -0.471 | -0.1697 | Yes |
| 176 | NME1 |  |  | 8573 | -0.471 | -0.1552 | Yes |
| 177 | POP7 |  |  | 8605 | -0.495 | -0.1434 | Yes |
| 178 | CDK4 |  |  | 8625 | -0.510 | -0.1298 | Yes |
| 179 | JPT1 |  |  | 8647 | -0.523 | -0.1160 | Yes |
| 180 | HMGA1 |  |  | 8675 | -0.543 | -0.1023 | Yes |
| 181 | CDKN1A |  |  | 8679 | -0.545 | -0.0858 | Yes |
| 182 | SNRPB |  |  | 8683 | -0.547 | -0.0692 | Yes |
| 183 | TUBB |  |  | 8763 | -0.621 | -0.0590 | Yes |
| 184 | DEK |  |  | 8770 | -0.627 | -0.0403 | Yes |
| 185 | STMN1 |  |  | 8783 | -0.638 | -0.0219 | Yes |
| 186 | RANBP1 |  |  | 8819 | -0.669 | -0.0052 | Yes |
| 187 | UBE2S |  |  | 8901 | -0.772 | 0.0094 | Yes |
Table: GSEA details [plain text format]

  

Fig 2: HALLMARK\_E2F\_TARGETS: Random ES distribution      
 Gene set null distribution of ES for **HALLMARK\_E2F\_TARGETS**
